# Epithelial thickness as a new predictor of recurrence in oral leukoplakia after photodynamic therapy: a retrospective cohort study

**DOI:** 10.1186/s12903-026-08832-3

**Published:** 2026-06-11

**Authors:** Wenxin Mu, Dongyao Wang, Qian Lu, Zhipei Chen, Linglan Yang, Xiaoan Tao

**Affiliations:** 1 Hospital of Stomatology, Guanghua School of Stomatology, Sun Yat-Sen University, Guangzhou, China; 2 Guangdong Provincial Clinical Research Center of Oral Diseases, Guangzhou, China

**Keywords:** Oral leukoplakia, Photodynamic therapy, Epithelial thickness, Initial response, Short-term response, Recurrence, Risk factors

## Abstract

**Background:**

Recent studies have reported encouraging clinical efficacy of photodynamic therapy (PDT) for oral leukoplakia (OLK), yet limitations remain, including inconsistent outcomes across centers, incomplete analysis indicators, and insufficient prognostic accuracy. This retrospective cohort study aimed to evaluate the efficacy of PDT for OLK and to explore potential influencing factors.

**Methods:**

A total of 60 patients with clinically and pathologically confirmed OLK were enrolled. All patients received one or more sessions of PDT with 5-aminolevulinic acid (5-ALA). Initial response was assessed at 1 ~ 3 weeks after the first session, and short-term efficacy was evaluated at 4 weeks after the final session. All patients were subsequently followed up to monitor lesion recurrence and malignant transformation. Univariate and multivariate logistic regression models were used to identify factors associated with treatment response. Kaplan–Meier survival analysis with log-rank test and Cox proportional hazards regression models were applied to analyze risk factors for recurrence.

**Results:**

Assessment after the first treatment demonstrated a mean lesion area reduction of 40.3%, with an initial total response (ITR) rate of 78.3%. After the final treatment, the mean reduction in lesion area was 74.4%. Complete response (CR), partial response (PR), and no response (NR) were achieved in 30.0%, 60.0%, and 10.0% of patients, respectively, resulting in a total response (TR) rate of 90.0%. During a mean follow-up of 10.8 months, recurrence occurred in 26 patients (48.1%), and two patients (3.3%) developed malignant transformation. Multivariate Cox analysis identified that lesions with epithelial thickness ≥ 400 μm (HR: 6.88, *p* = 0.005), non-smoking status (HR: 0.18,* p* = 0.025), and lesion location in a dangerous region (HR: 8.62, *p* = 0.027) were associated with an increased risk of recurrence.

**Conclusion:**

ALA-PDT is an effective treatment for OLK, demonstrating favorable efficacy in both initial and short-term response. Epithelial thickness is a novel predictor for recurrence risk, alongside established factors such as smoking history and lesion location in a dangerous region. Nevertheless, vigilant long-term monitoring remains essential due to a non-negligible rate of recurrence.

**Trial registration:**

chictr.org.cn, identifier ChiCTR2500099540 (March 25, 2025).

**Supplementary Information:**

The online version contains supplementary material available at 10.1186/s12903-026-08832-3.

## Background

Oral leukoplakia (OLK) is a clinically defined white patch or plaque of the oral mucosa that cannot be wiped off and cannot be classified as any other definable lesion based on clinical or histopathological features [1, 2]. It is considered one of the oral potentially malignant disorders (OPMDs) with a significant risk of malignant transformation, which has been reported to range from 0.13% to 34.0%, with an overall estimated rate of 3.5% [3–5]. Currently, the management of OLK remains challenging. Several treatment options are available—including pharmacotherapy, surgical excision, and laser ablation—yet none are considered curative, and recurrence remains a significant clinical challenge [6, 7].

Recently, photodynamic therapy (PDT) has emerged as a promising treatment option for OLK. PDT employs a photosensitizer and a specific light source in the presence of oxygen within biological tissues to selectively destroy abnormally proliferative tissue, offering advantages such as minimal invasiveness and high targeting precision [8]. This targeting precision is partly attributed to the Warburg effect in dysplastic cells (particularly neoplastic cells)—a metabolic hallmark characterized by elevated glycolytic activity that promotes the selective uptake and accumulation of photosensitizer precursors (e.g., 5-ALA) in lesional tissues, thereby enhancing precise destruction of abnormally proliferative cells while minimizing damage to the surrounding normal mucosa [9, 10]. It has been widely applied internationally in the treatment of premalignant and malignant lesions of the skin, digestive tract, and urogenital mucosa. In the field of oral medicine, PDT is primarily used for oral tumors and OPMDs, showing particular value in the treatment of OLK [10].

In recent years, numerous studies have reported the notable efficacy of PDT in treating OLK. However, considerable inconsistencies exist among different clinical centers, along with incomplete evaluation metrics and limited accuracy in prognostic assessment. One study found that PDT yields better outcomes for homogeneous OLK [11], whereas others reported superior efficacy for non-homogeneous OLK, lesions outside dangerous region, and those with moderate-to-severe dysplasia [12, 13]. Meanwhile, some studies indicated that factors such as patient gender, age, clinical type, lesion size, degree of dysplasia, and lesion location did not significantly influence the short-term efficacy of PDT [14]. Furthermore, available data consistently demonstrate a marked short-term treatment effect of PDT on OLK, with overall response rates ranging from 68 to 87% [11, 12, 14, 15]. Nevertheless, follow-up studies have revealed high rates of recurrence and malignant transformation after PDT, with substantial heterogeneity across different clinical centers. Therefore, it is essential to investigate the factors influencing disease recurrence and malignant progression. A thorough analysis of these factors will help manage clinician–patient expectations and provide a basis for personalized follow-up and treatment strategies following PDT.

As is well recognized, the primary pathological feature of OLK is epithelial hyperplasia accompanied by hyperkeratosis [16], which results in increased epithelial thickness. The efficacy of PDT is governed by several key determinants, notably the photosensitizer concentration and the optical properties of the target tissue [13]. Previous studies have proposed a similar hypothesis: the thicker keratin layer on the surface of OLK lesions can interfere with ALA diffusion and absorption while reducing light penetration to the underlying lesional cells. This, in turn, leads to unsatisfactory clinical outcomes [17]. Moreover, it has been reported that pretreatment with CO₂ laser, waterlase, or plum-blossom needle can enhance protoporphyrin IX (PpIX) accumulation and improve PDT outcomes [18–21], indirectly supporting the influence of epithelial thickness on treatment efficacy. Notably, however, epithelial thickness has not been systematically included as an observational parameter in previous studies. This omission may partly explain the inconsistent conclusions and limited accuracy in predicting therapeutic outcomes across different clinical centers. Therefore, we propose that incorporating epithelial thickness as an evaluation metric could help improve the prediction of PDT efficacy and provide a more reliable basis for clinical decision-making.

In clinical practice, patients with OLK typically undergo PDT once or multiple times [12, 14]. Previous studies have largely relied on the short-term response assessed after the final treatment session to reflect individual sensitivity to PDT and to predict long-term recurrence or malignant transformation. However, our clinical observations suggest that the initial treatment response varies considerably among OLK patients and may better reflect interindividual differences in sensitivity to PDT. The strategy of using early treatment response to adjust therapeutic regimens or predict long term outcomes is well-established in oncology and chronic disease management [22–24]. We propose that this approach can be adapted to PDT-based management of OLK. This study therefore focuses on initial treatment response as a key metric. Despite limited previous reporting in PDT for OLK, we consider it potentially valuable for predicting recurrence and malignant transformation, thus supporting personalized clinical decisions.

This study aimed to evaluate the efficacy of PDT in treating OLK and to analyze factors influencing treatment outcomes, with a specific focus on initial treatment response and epithelial thickness, in order to optimize clinical management strategies.

## Methods

This study is a single-center retrospective cohort study. The scientific and ethical protocols of the research have been approved by the Medical Ethics Committee of Hospital of Stomatology, Sun Yat-sen University (KQEC-2025–022-01). Prospective registration was completed on the ChiCTR system (ChiCTR2500099540) prior to the initiation of case screening. The reporting of this study adheres to the STROBE guidelines. This study was conducted in accordance with the Declaration of Helsinki, and written informed consent was obtained from each patient before treatment.

### Patients

Patients with oral leukoplakia who were diagnosed and treated at the Hospital of Stomatology, Sun Yat-sen University between April 2021 and September 2024 were enrolled in this study, totaling 60 subjects. The diagnostic criteria integrated the definitions of the Society of Oral Medicine, Chinese Stomatological Association and the World Health Organization [10, 25–27]. The diagnostic process can be summarized as follows: first, a clinical examination (inspection and palpation) was performed to exclude other definable oral conditions; second, after removal of suspected causative factors (such as smoking or mechanical irritation) for 2 ~ 4 weeks, if the lesion persisted, a biopsy was performed for histopathological confirmation. Histopathological confirmation of OLK was one of the inclusion criteria for this study. If there had been no progress in the condition within the past 2 years, the histopathological examination results within the past 2 years would be recognized, and the patient would have completed at least one course of PDT treatment. Additionally, patients were excluded from the cohort if they had a history of malignant tumors in the oral cavity or other parts of the body, or if they presented with incomplete clinicopathological information or follow-up records.

Prior to the initiation of treatment, we recorded the demographics of patients, including gender (female/male), age (years), duration of disease course (< 12 months/≥ 12months), smoking history (no/yes), drinking history (no/yes), lesion location (ventral tongue and floor of mouth/dorsum of the tongue/buccal/gingiva/palate), dangerous region (no/yes), total lesion area (< 200mm^2^/≥ 200mm^2^), clinical type (non-homogeneous/homogeneous), and epithelial dysplasia (no-mild/moderate-severe). In fact, we systematically evaluated multiple histopathological features (hyperkeratosis, acanthosis, inflammatory changes, epithelial thickness, etc.). Preliminary analyses showed that only epithelial thickness was significantly associated with recurrence (*p* = 0.030), as detailed in Table S1. Although epithelial dysplasia did not reach statistical significance in this cohort, it was retained based on literature consensus. Other features were not included in subsequent analyses due to lack of significant association. It is particularly important to note that the disease duration was defined as the time from the patient’s first noticing the lesion (self-reported onset). Among these, the dorsal and lateral surfaces of the tongue, ventral surface of the tongue, soft palate, retromolar trigone, and inner triangle of the angle of the lips are classified as the dangerous region of the oral mucosa, while other sites are considered non-dangerous regions.

**Fig. 1:**
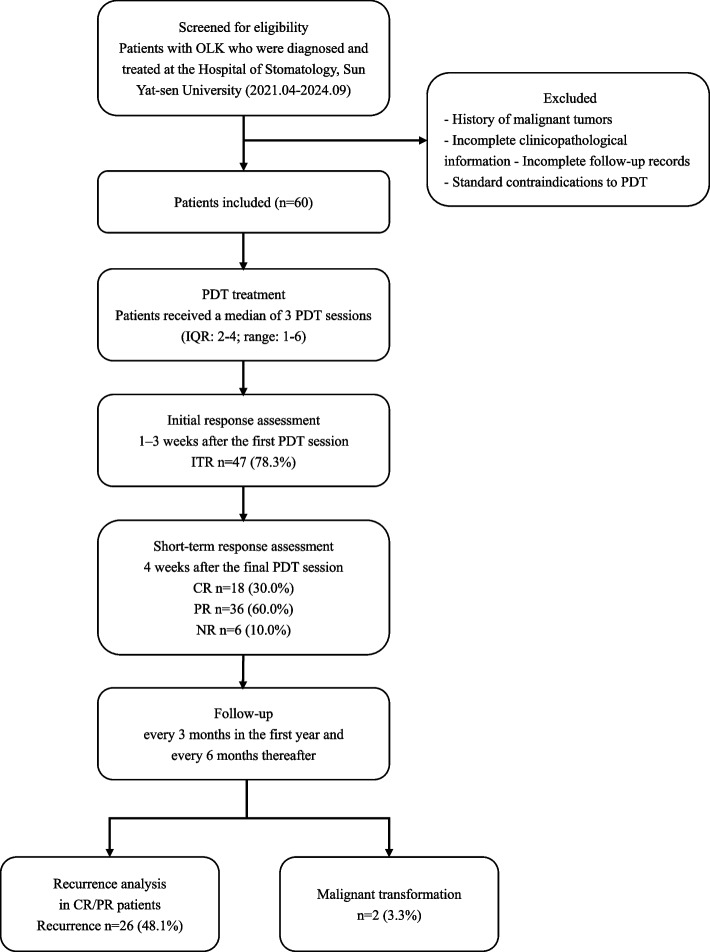
Study flowchart. OLK: oral leukoplakia; PDT: photodynamic therapy; IQR: interquartile range; ITR: initial total response; CR: complete response; PR: partial response; NR: no response

The overall study workflow, including patient screening, treatment, follow-up, and outcome assessment, is illustrated in the study flowchart (Fig. 1).

### Treatment procedure

PDT was performed as described previously [10]. In brief, the lesion was completely covered with a thin, sterile cotton gauze soaked in 20% 5-aminolevulinic acid (5-ALA) and incubated for 3 h to ensure adequate ALA penetration and protoporphyrin IX accumulation. Specifically, we used a commercially available, standardized pharmaceutical product, aminolevulinic acid hydrochloride topical powder (brand name: ALA, manufactured by Shanghai Fudan-Zhangjiang Bio-Pharmaceutical Co., Ltd., Shanghai, China). The product was stored protected from light, tightly sealed, and at a temperature not exceeding 20 °C. Each vial contained 118 mg of the active ingredient. Prior to each treatment session, the powder was freshly reconstituted with 0.5 mL sterile water for injection to obtain a 20% solution, which was applied topically to the lesion. The reconstituted solution was used within 4 h and applied to the lesion for no less than 3 h, with the entire procedure performed under light-protected conditions. To protect the photosensitizer from dilution by saliva, the ALA-soaked gauze was covered with a starch film, food-grade cling film, and thick gauze. Following this, to alleviate the burning or painful sensation during laser irradiation, local infiltration anesthesia was administered at the lesion site. A semiconductor laser (GYS-PDT-S2000, China Medical, 635 nm, 100 mW, continuous wave) with a wavelength of 630 ± 5 nm was used as the light source for irradiation. The power density was set at 100 mW/cm^2^, and each treatment area received 1,000 s of irradiation to achieve an energy density of 100 J/cm^2^. Treatments were repeated at intervals of 2 to 3 weeks. In this study, CO₂ laser was applied as a pretreatment in selected patients, particularly those with pronounced hyperkeratosis, to remove the keratin layer before photosensitizer application. This approach was intended to enhance drug penetration and light penetration to the basal layer, thereby improving the efficacy of PDT. The procedure was performed after local infiltration anesthesia and before the application of ALA. The CO₂ laser parameters were as follows: wavelength 10,600 nm, average power 0.81 W, peak power 1.62 W, gated mode, spot size 0.8 mm, and irradiation time ranging from 1–6 min depending on the lesion size. The decision to use CO₂ laser was based on the clinician’s clinical experience and judgment.

### Clinical evaluation and follow-up

The assessment criteria were as follows [10]: Compared to pre-treatment, if the visible lesion(s) had completely disappeared, it was defined as complete response (CR). If the lesion(s) had not completely disappeared but the area reduction was ≥ 20%, it was defined as partial response (PR). If the lesion area reduction was < 20% or the lesion area had increased, it was defined as no response (NR). The initial efficacy was assessed 1 ~ 3 weeks after the first PDT session. Initial total response (ITR) rate was defined as the percentage of patients showing complete disappearance of the lesion or a reduction in lesion area ≥ 20% relative to the total cohort size. The short-term efficacy was assessed 4 weeks after the final PDT session. Total response (TR) rate was defined as the percentage of patients achieving CR or PR relative to the entire cohort, and the CR rate was defined as the percentage of patients achieving CR relative to the entire cohort.

All patients were entered into a follow-up program with clinical assessments every three months in the first year and every six months from the second year onward. The objective was to monitor for recurrence and malignant transformation in CR/PR patients, whereas for NR patients, the focus was solely on detecting malignant transformation. Clinical recurrence and malignant transformation were assessed at each follow-up visit. Recurrence was defined as: 1) the reappearance of white lesions during follow-up in patients who had achieved CR at the 4-week assessment after the final PDT session, or 2) an increase in lesion area of ≥ 20% during follow-up in patients who had achieved PR at the 4-week assessment after the final PDT session. During follow-up, experienced clinicians comprehensively assessed the lesions based on clinical symptoms and multiple factors including area, color, and texture. If a lesion exhibited signs suggestive of potential malignant transformation, a biopsy was performed. Based on routine clinical practice and literature consensus, such signs typically included induration, ulceration, erythroplakia, rapid increase in lesion size, surface granularity or verrucous change, bleeding, spontaneous pain, and fixation to underlying tissue [28, 29]**.** Malignant transformation (MT) was defined as the presence of cancerous cells upon histopathological examination of the biopsy specimen. If MT occurred at any point during the study, follow-up was terminated immediately, and appropriate treatment was arranged.

### Sample size calculation

This study is a single-center, non-randomized controlled, single-group paired, self-controlled before-after retrospective study. The primary outcome was the "Percentage Reduction in Area (%RA)", measured at 4 weeks after the final treatment session. Based on previous research data, the mean %RA for PDT-treated OLK was 0.666, with a standard deviation was 0.327 [30]. In clinical practice and related studies on oral leukoplakia treatment, a "50% reduction in leukoplakia area" is often used as the criterion for treatment efficacy [31–33]. Therefore, this study set the efficacy threshold at 0.5. As %RA is a superiority indicator, with a one-sided α = 0.025 and a power of 90%, the minimum sample size calculated using PASS 15.0.5 (NCSS, LLC. Kaysville, UT, USA) software was 41. Since this is a retrospective study, loss to follow-up and withdrawal of informed consent were not considered. Thus, a minimum of 41 subjects were required. Given that this study will also explore independent influencing factors that affect initial efficacy, short-term efficacy, recurrence, and malignancy, and in order to analyze the differences between subgroups in depth, all 60 subjects who meet the inclusion and exclusion criteria were ultimately included. This ensures sufficient sample size and enhances the reliability of the results.

### Measurement of epithelial thickness

**Fig. 2:**
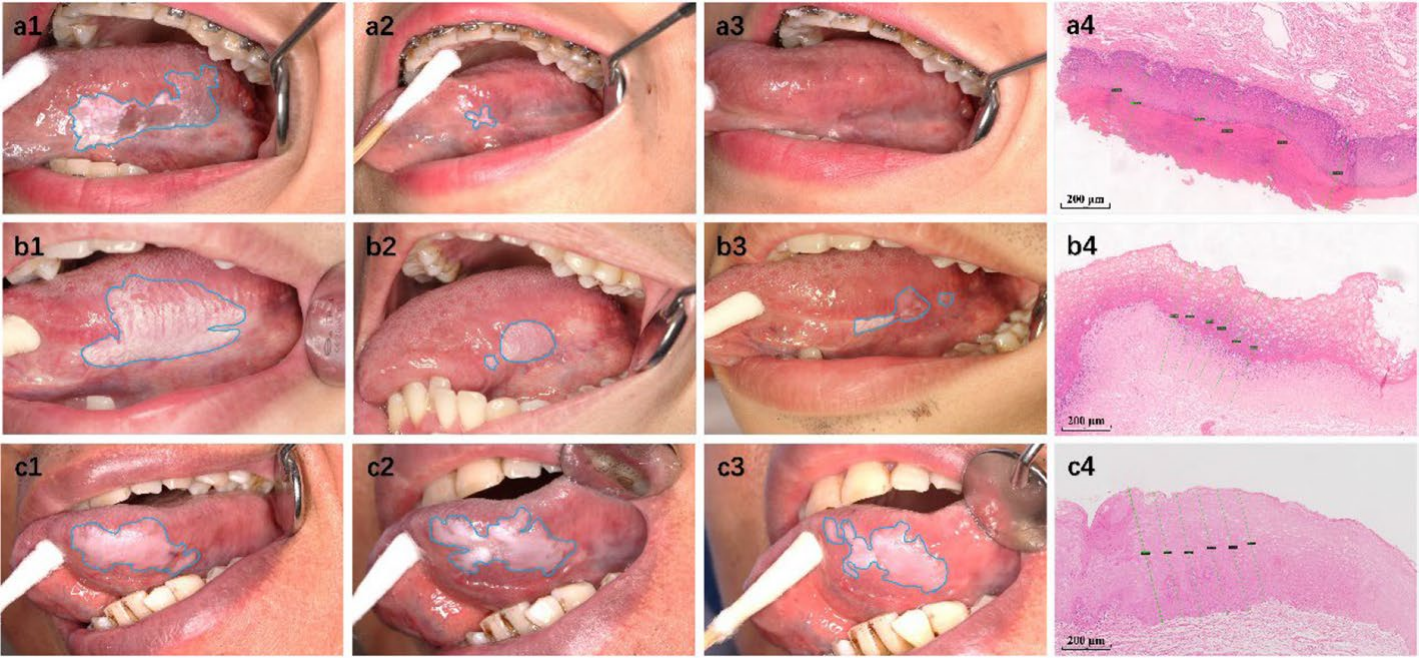
Representative pictures showing the different clinical outcomes. Complete Response (CR; a1-a4), Partial Response (PR; b1-b4), and No Response (NR; c1-c4) assessed 4 weeks after the final treatment session. For all groups, a1, b1, and c1 show the pre-treatment condition; a2, b2, and c2 were taken two weeks after the initial treatment; a3, b3, and c3 were taken two weeks after the final treatment; and a4, b4, and c4 are corresponding pathological sections (H&E staining) showing mild dysplasia

The tissues used for H&E staining and epithelial thickness measurement were obtained from the initial diagnostic biopsies. No additional biopsies were performed for research purposes. The interval between biopsy and the first PDT session was within 2 years, and most patients received treatment shortly after diagnosis. HE-stained sections of OLK tissues from the subjects were used to measure epithelial thickness. The slides were scanned using a slide scanner (Leica Aperio AT2, Leica Microsystems, Wetzlar, Germany) for digital image analysis. The images were measured blindly by two certified anatomic pathologists. Epithelial thickness was measured on HE-stained sections using the image analysis software Aperio ImageScope (Version 12.4.3; Leica Microsystems). For each slide, five representative 200 × fields were selected. As illustrated in Fig. 2 (a4, b4, c4), measurements were taken from the stratum basale to the stratum corneum (in keratinized epithelium) or to the stratum superficiale (in non-keratinized epithelium). Each field was measured five times, and the average value was calculated to minimize measurement error, thereby obtaining the epithelial thickness.

### Statistical analysis

Data were analyzed using SPSS version 20 (SPSS Inc., Chicago, IL, USA). Quantitative data were summarized as mean, median, and standard deviation (SD). Categorical data were described using frequencies and percentages. A one-sample t-test was used to evaluate the effectiveness of PDT in treating OLK. Independent factors associated with overall efficacy were examined using binary logistic regression. Independent factors influencing recurrence were analyzed with Kaplan–Meier survival curves and Cox proportional hazards regression models. A *p* value of 0.05 or less was considered statistically significant.

## Results

### Patient characteristics

Based on the inclusion and exclusion criteria, 60 subjects were enrolled in this study. Their clinical and histological characteristics are summarized in Table 1. Although the retromolar trigone, soft palate, and inner triangle of the angle of the lips are anatomically defined as dangerous regions, no lesions were located at these specific subsites in our cohort. Accordingly, all buccal, gingival, and palatal lesions were classified as non-dangerous regions. Notably, only one subject reported regular alcohol consumption; all others were classified as having no drinking history, including both "never drinking" and "occasional drinking". Due to this extreme sparsity, drinking history was excluded from all regression models to avoid statistical instability. Additionally, the distribution of patients across grades of epithelial dysplasia was highly unbalanced: no dysplasia in 2 (3.3%), mild in 15 (25.0%), moderate in 31 (51.7%), and severe in 12 (20.0%). This distribution reflects clinical biopsy practices—clinicians tend to recommend invasive biopsy only when dysplasia is suspected, resulting in a very small number of non-dysplastic cases. To avoid low statistical power and potential masking of other variables in multivariate analyses, we combined the grades into no-mild versus moderate-severe (binary variable) for subsequent analyses, following recent literature [11, 12, 15]. The ITR, short-term efficacy, and recurrence outcomes by individual dysplasia grade are presented in Table S2 for potential use in future evidence syntheses.

**Table 1:** Demographics of patients who received photodynamic therapy

Variable	n (%) or mean ± SD
Overall (*n* = 60)	
Gender	
Female	28 (46.7%)
Male	32 (53.3%)
Age (year)	52.5 ± 14.3
Duration of disease course (months)	21.0 ± 31.7
< 12 months	35 (58.3%)
≥ 12 months	25 (41.7%)
Smoking history	
No	43 (71.7%)
Yes	17 (28.3%)
Drinking history	
No	59 (98.3%)
Yes	1 (1.7%)
Lesion location	
Ventral tongue and floor of mouth	43 (71.7%)
Dorsum of the tongue	3 (5.0%)
Buccal^a^	8 (13.3%)
Gingiva^b^	3 (5.0%)
Palate^c^	3 (5.0%)
Dangerous region	
No	14 (23.3%)
Yes	46 (76.7%)
Total lesion area (mm^2^)	205.9 ± 140.8
< 200 mm^2^	23 (38.3%)
≥ 200 mm^2^	37 (61.7%)
Clinical type	
Non-homogenous	23 (38.3%)
Homogenous	37 (61.7%)
Epithelial dysplasia	
No	2 (3.3%)
Mild	15 (25.0%)
Moderate	31 (51.7%)
Severe	12 (20.0%)

^a^No buccal lesions were located in the inner triangle of the angle of the lips; therefore, all 8 cases were classified as non-dangerous region

^b^No gingival lesions were located in the retromolar trigone; thus, all 3 cases were classified as non-dangerous region

^c^No palatal lesions were located in the soft palate; thus, all 3 cases were classified as non-dangerous region

Furthermore, digitally scanned whole-slide images were available for 53 subjects, whose epithelial thickness was measured as 482.6 ± 209.0 μm, and the median was 469 μm, which is generally consistent with previous studies [34, 35]. Among them, 31 cases had an epithelial thickness ≥ 400 μm, and 22 cases had an epithelial thickness < 400 μm. The remaining 7 cases, for which only written pathology reports (including the degree of dysplasia) were available without original whole-slide images, precluding accurate epithelial thickness measurement, were therefore excluded from epithelial thickness-related analyses.

It should be noted that, in routine clinical practice, all patients underwent systematic screening for standard contraindications to PDT-including porphyria, photosensitivity disorders, and severe hepatic disease-prior to treatment, in accordance with established clinical protocols. Patients with any such contraindications would not have received PDT.

### Clinical outcomes

The number of PDT sessions was determined individually based on lesion response and patient preference. Patients received a median of 3 PDT sessions (IQR: 2–4; range: 1–6). Treatment was continued until complete lesion clearance was achieved, no further response was observed after an additional session, or the patient opted to discontinue due to satisfactory outcomes [12]. The primary outcome of this study was the percentage reduction in area (%RA), defined as the percentage decrease in lesion area measured 4 weeks after the final PDT session compared to the pre-treatment lesion area. The %RA in this study was 74.4% (SD: 0.289; 95% CI: 0.669–0.819). With a pre-specified target value of 0.5 [31–33] and %RA being a higher-is-better indicator, the lower limit of the 95% confidence interval (0.669) exceeded the target value of 0.5, indicating that PDT was significantly effective in treating OLK.

Based on clinical records and pathological sections, and synthesizing previous studies and our clinical experience, we selected ten potential predictors for treatment response and recurrence. These included age, gender, duration of disease course, smoke, dangerous region, total lesion area, epithelial thickness, clinical type, epithelial dysplasia, CO_2_ laser therapy. Additionally, ITR and CR were included in the analysis of risk factors for recurrence and malignant transformation.

### ITR and influencing factors

The initial treatment response was assessed at 1 ~ 3 weeks following the initial treatment, showing a mean reduction in the white lesion area of OLK by 40.3% (0.403 ± 0.224). The ITR rate was 78.3%. Univariate and multivariate logistic regression analyses were performed to identify variables influencing ITR. Of the 10 variables investigated, none demonstrated a statistically significant association with ITR in either univariate or multivariate analyses (all *p* > 0.05), as detailed in Table 3. Notably, although not statistically significant, several factors showed potentially meaningful effects. Lesions with an epithelial thickness ≥ 400 μm were more likely to achieve a favorable initial treatment response compared to those below this threshold (*p* = 0.139; OR: 2.70). After adjusting for other covariates in the multivariate model, the association between epithelial thickness and ITR strengthened but did not reach statistical significance (*p* = 0.092; OR [95% CI]: 3.63 [0.81–16.2]), suggesting that epithelial thickness may represent a potential influencing factor for ITR. Patients with moderate-to-severe epithelial dysplasia *(p* = 0.350; OR [95% CI]: 2.26 [0.41–12.5]) and non-smokers (*p* = 0.244; OR [95% CI]: 0.39 [0.08–1.90]) also showed higher odds of favorable initial treatment response, although these results were not statistically significant and may be attributable to limited statistical power. Of note, since the OR was approximately 1.0 with a non-significant p-value in the univariate analysis for variables such as age and gender, these were not included in the multivariate model to enhance its stability.

**Table 2:** Influencing factors of ITR after PDT (*N* = 53^a^)

	**ITR**	**Univariate analysis**		**Multivariate analysis**	
		**OR [95% CI]**	***P* value**	**OR [95% CI]**	***P* value**
Age (year)		0.99 [0.95–1.05]	0.980	NA^b^	NA^b^
Gender					
Female	18/23 (78.3%)	Ref			
Male	23/30 (76.7%)	0.91 [0.25–3.36]	0.891	NA^b^	NA^b^
Duration of disease course (months)					
< 12 months	24/32 (75.0%)	Ref		Ref	
≥ 12 months	17/21 (81.0%)	1.42 [0.37–5.47]	0.613	1.98 [0.37–10.6]	0.427
Smoking history					
No	31/37 (83.8%)	Ref		Ref	
Yes	10/16 (62.5%)	0.32 [0.09–1.23]	0.097	0.39 [0.08–1.90]	0.244
Dangerous region					
No	10/14 (71.4%)	Ref		Ref	
Yes	31/39 (79.5%)	1.55 [0.38–6.26]	0.538	1.11 [0.18–6.81]	0.910
Total lesion area (mm^2^)					
< 200 mm^2^	25/31 (80.6%)	Ref		Ref	
≥ 200 mm^2^	16/22 (72.7%)	0.64 [0.18–2.33]	0.499	0.52 [0.09–2.90]	0.459
Epithelial thickness (μm)					
< 400 μm	14/22 (63.6%)	Ref		Ref	
≥ 400 μm	27/31 (87.1%)	2.70 [0.72–10.1]	0.139	3.63 [0.81–16.2]	0.092
Clinical type					
Non-homogenous	25/34 (73.5%)	Ref		Ref	
Homogenous	16/19 (84.2%)	1.92 [0.45–8.18]	0.378	0.85 [0.13–5.62]	0.863
Epithelial dysplasia					
No-Mild	9/14 (64.3%)	Ref		Ref	
Moderate- Severe	32/39 (82.1%)	2.54 [0.65–9.95]	0.181	2.26 [0.41–12.5]	0.350
CO_2_ laser					
No	31/37 (83.8%)	Ref		Ref	
Yes	10/16 (62.5%)	0.32 [0.09–1.23]	0.097	0.35 [0.07–1.85]	0.217

*ITR* initial total response, *OR* Odds Ratio, *95%CI* 95% Confidence Interval

^a^the sample size for the epithelial thickness model is *n* = 53 after listwise deletion of 7 cases with missing values

^b^OR was approximately 1.0 with a non-significant *P* value in univariate analysis and was therefore not included in the multivariate model

### TR and influencing factors

At 4 weeks after the final treatment session, 30.0% (18/60) of patients achieved CR, 60.0% (36/60) achieved PR, and 10.0% (6/60) showed NR (Fig. 2). The TR rate was 90.0% (54/60). To identify factors influencing TR, univariate and multivariate logistic regression analyses were conducted to evaluate variables associated with the efficacy of PDT for OLK. None of the 10 potential factors showed a statistically significant association with TR in the univariate analysis and multivariate analysis (all *p* > 0.05). It should be noted that in the multivariate analysis, the variable of age was excluded due to an OR approximating 1.0 with a non-significant p-value in univariate analysis. Epithelial thickness, however, was retained as a predefined variable of interest. Detailed results are provided in Table 3**.** Nevertheless, several variables demonstrated suggestive, though statistically non-significant, associations with TR in the multivariate analysis. For instance, patients with a duration ≥ 12 months (*p* = 0.170; OR [95% CI]: 6.94 [0.44–110]), total lesion area < 200 mm^2^ (*p* = 0.281; OR [95% CI]: 0.20 [0.01–3.77]), without smoking history (*p* = 0.327; OR [95% CI]: 0.26 [0.02–3.81]), moderate-severe epithelial dysplasia lesion (*p* = 0.350; OR [95% CI]: 3.83 [0.23–63.6]), non-homogeneous lesions (*p* = 0.426; OR [95% CI]: 0.36 [0.03–4.41]), lesions located in non-dangerous regions (*p* = 0.523; OR [95% CI]: 0.39 [0.02–7.16]), and epithelial thickness ≥ 400 μm *(p* = 0.527; OR [95% CI]: 2.11 [0.21–21.4]) had higher odds of achieving treatment response. However, none of these associations reached statistical significance, and the wide confidence intervals reflect substantial uncertainty, likely due to limited statistical power.

**Table 3:** Influencing factors of TR after PDT (*N* = 53^a^)

Characteristic	TR	Univariate analysis		Multivariate analysis	
		OR [95% CI]	*P* value	OR [95% CI]	*P* value
Age (year)		0.99 [0.92–1.06]	0.677	NA^b^	NA^b^
Gender					
Female	22/23 (95.7%)	Ref		Ref	
Male	26/30 (86.7%)	0.30 [0.03–2.84]	0.291	0.64 [0.03–15.5]	0.782
Duration of disease course (months)					
< 12 months	28/32 (87.5%)	Ref		Ref	
≥ 12 months	20/21 (95.2%)	2.86 [0.30–27.5]	0.364	6.94 [0.44–110]	0.170
Smoking history					
No	35/37 (94.6%)	Ref		Ref	
Yes	13/16 (81.2%)	0.25 [0.04–1.65]	0.150	0.26 [0.02–3.81]	0.327
Dangerous region					
No	13/14 (92.9%)	Ref		Ref	
Yes	35/39 (89.7%)	0.67 [0.07–6.59]	0.734	0.39 [0.02–7.16]	0.523
Total lesion area (mm^2^)					
< 200 mm^2^	29/31 (93.5%)	Ref		Ref	
≥ 200 mm^2^	19/22 (86.4%)	0.44 [0.07–2.86]	0.388	0.20 [0.01–3.77]	0.281
Epithelial thickness (μm)					
< 400 μm	19/21 (90.5%)	Ref			
≥ 400 μm	29/32 (90.6%)	1.02 [0.16–6.67]	0.986	2.11 [0.21–21.4]	0.527
Clinical type					
Non-homogenous	31/34 (91.2%)	Ref		Ref	
Homogenous	17/19 (89.5%)	0.82 [0.13–5.42]	0.839	0.36 [0.03–4.41]	0.426
Epithelial dysplasia					
No-Mild	12/14 (85.7%)	Ref		Ref	
Moderate- Severe	36/39 (92.3%)	2.00 [0.30–13.4]	0.476	3.83 [0.23–63.6]	0.350
CO_2_ laser					
No	34/37 (91.9%)	Ref		Ref	
Yes	14/16 (87.5%)	0.62 [0.09–4.11]	0.618	0.87 [0.08–9.89]	0.907

*TR* total response, *OR* Odds Ratio, *95%CI* 95% Confidence Interval

^a^the sample size for the epithelial thickness model is *n* = 53 after listwise deletion of 7 cases with missing values

^b^OR was approximately 1.0 with a non-significant *P* value in univariate analysis and was therefore not included in the multivariate model

### Recurrence and risk factors

**Fig. 3:**
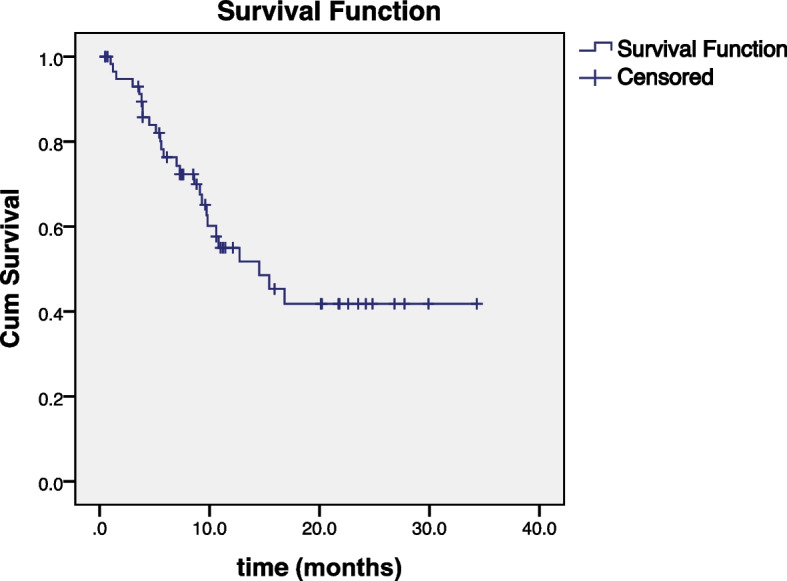
Kaplan–Meier survival curves for recurrence in OLK patients following PDT

**Fig. 4:**
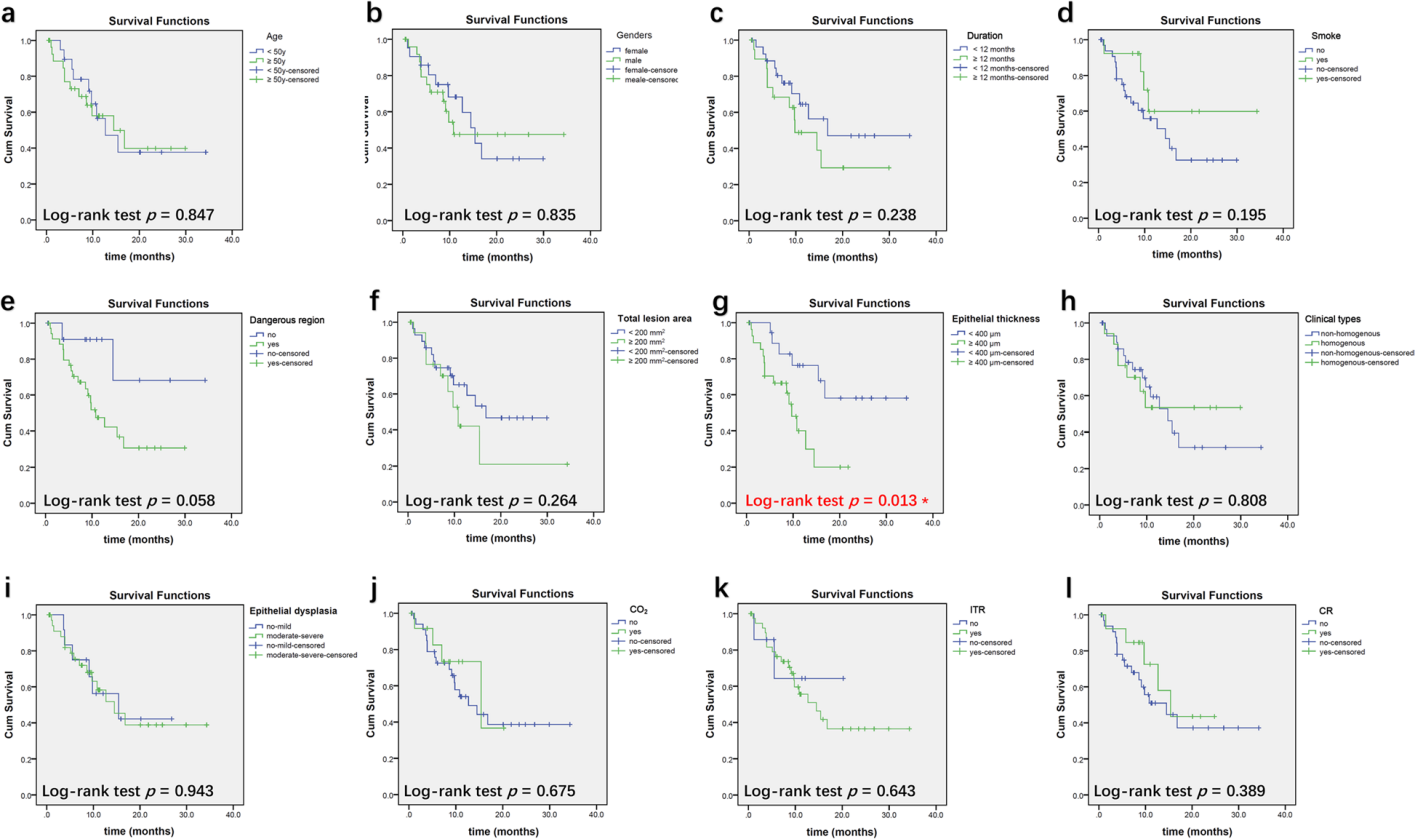
Comparisons of the recurrence of OLK after PDT in different patient groups. **a** Comparison of age groups between < 50 and ≥ 50 years. **b** Comparison between male and female. c Comparison of the duration of disease course between < 12 and ≥ 12 months. **d** Comparison between non-smokers and smokers. **e** Comparison between lesions located in dangerous region and non-dangerous region. **f** Comparison of total lesion area between < 200 and ≥ 200 mm^2^. **g** Comparison of lesions with epithelial thickness between < 400 and ≥ 400 μm. **h** Comparison between homogeneous and non-homogeneous lesions. **i** Comparison between no-mild and moderate-severe epithelial dysplasia. **j** Comparison between lesions treated with and without CO_2_ laser. **k** Comparison between patients achieving and not achieving ITR. **l** Comparison between patients achieving PR and CR. * a significant statistical difference was observed, *p* < 0.05

All 60 patients completed follow-up, with a mean follow-up time of 10.8 ± 8.1 months (range: 0.5 ~ 34.3 months). Among the 54 patients who achieved CR or PR, recurrence occurred in 26 cases (48.1%). The mean time to recurrence was 7.0 ± 4.1 months (range: 1.0 ~ 16.8 months). Kaplan–Meier survival analysis estimated the recurrence risk at 23.7% by 6 months and 45.0% by 1 year, with a median recurrence-free survival of approximately 14.5 months (Fig. 3). The NR patients (*n* = 6) were excluded from the analysis of risk factors for recurrence, as recurrence is not applicable to this group, and they received distinctly different subsequent treatments (primarily CO_2_ laser ablation or surgical excision) compared to the CR/PR patients (who received no further therapy or low-dose medication maintenance). We evaluated the influence of 12 variables on recurrence. An initial exploration of potential factors affecting recurrence was conducted using the Kaplan–Meier survival analysis, with the results presented in Fig. 4. As shown, only epithelial thickness demonstrated a statistically significant association with recurrence (log-rank test* p* = 0.013). Further univariate and multivariate Cox proportional hazards regression analyses were performed, with detailed results summarized in Table 4. In the univariate Cox regression analysis, epithelial thickness was the only factor significantly associated with recurrence (as a risk factor). Lesions with an epithelial thickness ≥ 400 μm had a higher recurrence rate compared to the reference group (*p* = 0.018; HR: 3.28; 95%CI: 1.23–8.76). For the multivariate Cox regression analysis, the variable age was excluded due to a non-significant result in the univariate analysis (with *p* value and HR close to 1). The remaining 11 variables were included in the multivariate Cox model. The analysis revealed that, in addition to epithelial thickness, which remained significantly associated with recurrence (*p* = 0.005, HR: 6.88, 95%CI: 1.80–26.4), smoking history and dangerous regions also became significant after multivariate adjustment. Specifically, non-smokers (*p* = 0.025, HR: 0.18, 95%CI: 0.04–0.81) and lesions located in dangerous regions (*p* = 0.027, HR: 8.62; 95%CI: 1.27–58.3) were associated with a significantly higher recurrence risk. Two patients developed malignant transformation, corresponding to a cumulative malignant transformation rate of 3.3% (2/60). The clinical and pathological characteristics of malignant transformation patients are presented in Table 5.

**Table 4:** Influencing factors of recurrence rate after PDT (*N* = 48^a^)

Characteristic	recurrence rate	Univariate analysis		Multivariate analysis	
		**OR (95% CI)**	***P* value**	**OR (95% CI)**	***P* value**
Age (year)		1.02 [0.99–1.05]	0.275	NA^b^	NA^b^
Gender					
Female	10/22 (45.5%)	Ref		Ref	
Male	11/26 (42.3%)	1.10 [0.46–2.59]	0.836	2.00 [0.59–6.74]	0.263
Duration of disease course (months)					
< 12 months	10/28 (35.7%)	Ref		Ref	
≥ 12 months	11/20 (55.0%)	1.67 [0.71–3.93]	0.244	2.16 [0.66–7.07]	0.205
Smoking history					
No	17/35 (48.6%)	Ref		Ref	
Yes	4/13 (30.8%)	0.49 [0.17–1.47]	0.205	0.18 [0.04–0.81]	0.025 ^*^
Dangerous region					
No	2/13 (15.4%)	Ref		Ref	
Yes	19/35 (54.3%)	3.71 [0.86–16.0]	0.078	8.62 [1.27–58.3]	0.027 ^*^
Total lesion area (mm^2^)					
< 200 mm^2^	12/29 (41.4%)	Ref		Ref	
≥ 200 mm^2^	9/19 (47.4%)	1.65 [0.68–3.99]	0.270	0.78 [0.21–2.92]	0.716
Epithelial thickness (μm)					
< 400 μm	6/19 (31.6%)	Ref		Ref	
≥ 400 μm	15/29 (51.7%)	3.28 [1.23–8.76]	0.018^*^	6.88 [1.80–26.4]	0.005^**^
Clinical type					
Non-homogenous	14/31 (45.2%)	Ref		Ref	
Homogenous	7/17 (41.2%)	0.89 [0.36–2.22]	0.808	0.32 [0.08–1.20]	0.091
Epithelial dysplasia					
No-Mild	6/12 (50.0%)	Ref			
Moderate- Severe	15/36 (41.7%)	1.04 [0.40–2.68]	0.943	NA ^b^	NA ^b^
CO_2_ laser					
No	17/34 (50.0%)	Ref		Ref	
Yes	4/14 (28.6%)	0.79 [0.26–2.37]	0.676	1.01 [0.20–5.00]	0.993
ITR					
No	2/6 (33.3%)	Ref			
Yes	14/32 (43.8%)	0.99 [0.466–2.10]	0.975	NA^b^	NA^b^
CR					
No	13/28 (46.4%)	Ref			
Yes	3/10 (30.0%)	0.53 [0.15–1.87]	0.325	0.41 [0.08–2.14]	0.290

*OR* Odds Ratio, *95%CI* 95% Confidence Interval, *ITR* initial total response, *CR* complete response

^⁎^asignificant statistical difference was observed, *P* < 0.05

^**^a significant statistical difference was observed, *P* < 0.01

^a^final *n* = 48 for recurrence analysis after excluding 5 NR patients missing follow-up from the analytic cohort (*n* = 53)

^b^OR was approximately 1.0 with a non-significant *P* value in univariate analysis and was therefore not included in the multivariate model

**Table 5:** Clinicopathological parameters of patients with malignant transformation

Gender	Age (year)	Smoking history	Drinking history	Lesion location	Dangerous region	Total lesion area(mm^2^)	Epithelial thickness (μm)	Clinical type	Dysplastic pathology	Malignant transformation time (months)	CO_2_ laser	ITR	TR
Female	57	No	No	buccal	No	147.0	507.6	Homogenous	Severely dysplastic	7.3	No	Yes	Yes
Male	49	Yes	No	Ventral of the tongue	Yes	646	132.8	Non-homogenous	Mild dysplastic	3.8	Yes	No	No

## Discussion

This study included 60 subjects who underwent one or more PDT sessions, resulting in a 74.4% reduction in white lesion area. TR rate of 90% and CR rate of 30% were achieved, which is encouraging and further supports the considerable potential of PDT in treating OLK and are consistent with the findings reported in prior literature [14, 36].

In analyzing factors affecting TR, neither univariate nor multivariate analysis identified any significant predictors, which aligns with findings from Jing et al. [14] and Chen et al. [17]. However, other studies report conflicting results. For example, Wang et al. [15] associated non-homogeneous type and moderate-to-severe epithelial dysplasia with higher TR, while Pietruska et al. [37] found PDT more effective for cheek and lip lesions than those on the tongue or gums. Given PDT’s selective cytotoxicity for cancerous and dysplastic cells, it should theoretically be more effective in lesions with higher malignant potential. Our study also observed higher TR rates in cases with clinical features including duration ≥ 12 months, no smoking history, moderate-to-severe epithelial dysplasia, non-homogeneous type, or epithelial thickness ≥ 400 μm, although none of these associations reached statistical significance—likely due to limited sample size, a common constraint in such studies. Future large-scale studies are needed to strengthen the evidence, a goal of our ongoing work.

Unfortunately, ITR did not demonstrate satisfactory significant value in predicting recurrence/malignant transformation as we had anticipated, nor did it reveal significantly different outcomes across patient subgroups with different clinicopathological characteristics. However, we observed that the influence of epithelial thickness on ITR was relatively notable among various factors. Lesions with epithelial thickness ≥ 400 μm were more likely to achieve a favorable initial response (87.1 vs. 63.6%; *p* = 0.092; OR: 3.63). In other words, lesions with epithelial thickness ≥ 400 μm were 3.63 times more likely to achieve ITR, even though the difference did not reach statistical significance. This may be attributed to the non-random application of preoperative CO_2_ laser, as mentioned earlier. In our study, a higher proportion of patients with thicker keratin layers underwent combined CO_2_ laser treatment, which might have contributed to the better initial outcomes. Certainly, the limited sample size is also an evident contributing factor.

Recurrence and malignant transformation of OLK are persistent clinical concerns, with recurrent lesions carrying a heightened risk of oral cancer development [4, 21], constituting a key management challenge. Our study, over a mean follow-up of 10.8 months, found cumulative rates of 48.1% for recurrence and 3.3% for malignant transformation. Kaplan–Meier estimates further indicated a 1-year recurrence risk of 45% and a median recurrence-free survival of 14.5 months, aligning with existing research [11, 36, 38]. Previous studies have reported widely varying recurrence rates. A meta-analysis encompassing 16 studies with a total of 352 patients, who were followed for 1 to 119 months, found cumulative recurrence rates ranging from 0 to 60% [36]. The substantial heterogeneity in these cumulative recurrence rates is primarily attributable to differences in follow-up duration. Other contributing factors include variations in PDT parameters, the type and administration of photosensitizers, patient inclusion and exclusion criteria, and postoperative maintenance medication regimens. One study reported a recurrence rate of 27.1% within a six-month period after ALA-PDT treatment for OLK [38], which is largely consistent with our findings. Similarly, a study by Wang et al., with a mean follow-up of 20.3 months, observed a malignant transformation rate of 4% following ALA-PDT [11], also aligning closely with the results of our study.

In the analysis of factors influencing OLK recurrence after PDT, we notably found that the variable of epithelial thickness demonstrated significant predictive power for recurrence in Kaplan–Meier survival analysis, as well as in both univariate and multivariate Cox regression analyses, emerging as a risk factor for recurrence. This represents the most innovative finding of our study. Specifically, in the multivariate Cox analysis, we observed that lesions with epithelial thickness ≥ 400 μm had a significantly higher recurrence rate compared to the control group, with a hazard ratio of 6.88 (*p* = 0.005, HR: 6.88, 95% CI: 1.80–26.4). After adjustment via Kaplan–Meier survival analysis, we found that the 6-month postoperative recurrence risk was 33.5% in the epithelial thickness ≥ 400 μm group, with a 1-year recurrence risk of 60.0%. In contrast, the epithelial thickness < 400 μm group exhibited a 6-month recurrence risk of 11.5% and a 1-year recurrence risk of 23.7%. The proposed mechanism is that dysplastic cells reside primarily in the basal layer, requiring nearly full epithelial penetration by both light and photosensitizer, which leads to inadequate fluence and drug concentration at the target site [17]. Additionally, a thickened epithelium may create an altered redox microenvironment with upregulated antioxidant defenses (e.g., glutathione and Nrf2), which can scavenge reactive oxygen species generated by PDT, thereby further diminishing therapeutic efficacy and promoting residual cell survival [39–42]. Consequently, residual tumor-initiating cells may persist after PDT [13]. However, treatment is often concluded once the superficial lesion has clinically resolved [10], allowing these residual cells to escape destruction and ultimately cause recurrence. These findings support combining PDT with adjuvant therapies—such as CO₂ laser ablation, maintenance medication, or redox‑modulating agents that suppress antioxidant defenses (e.g., Nrf2 inhibitors or glutathione depletors)—particularly for lesions with epithelial thickness ≥ 400 μm, to enhance eradication and reduce recurrence.

Furthermore, multivariate Cox analysis revealed that after adjustment, smokers had a significantly lower recurrence risk than non-smokers (HR: 0.18, *p* = 0.025), indicating a higher risk in non-smokers. Simultaneously, lesions in dangerous region were associated with an 8.62-fold increase in recurrence risk (HR: 8.62, *p* = 0.027), likely because the contributing factor of friction and biochemical irritation persists [43]. Most previous studies have indicated that leukoplakia in non-smokers (idiopathic leukoplakia) and lesions in dangerous region is associated with higher-grade dysplasia, an increased risk of malignant transformation, and a poorer prognosis [44–46]. Consequently, clinicians should adopt comprehensive management strategies both before and after PDT in these patients. Pre-treatment measures should address both physical and chemical irritants, including the removal of sharp residual roots and crowns as well as defective prostheses, along with counseling on cessation of smoking and betel quid chewing. Post-treatment management should consist of adjunctive pharmacotherapy and intensified follow-up monitoring to minimize the likelihood of disease recurrence. We also noted that while a favorable initial response to PDT was associated with a higher recurrence risk in the univariate analysis (HR: 1.41, *p* = 0.646), this effect reversed in the adjusted multivariate Cox model, where ITR patients showed a lower recurrence risk—only 31% of that in non-ITR patients (HR: 0.31, *p* = 0.231). Although not statistically significant, this finding may still suggest a potential role of ITR in early prognostication, which could help guide the timely adjustment of treatment plans and patient expectations toward more personalized therapy.

Notably, the seemingly favorable initial response in thick lesions (≥ 400 μm) may be partly attributable to rapid surface desquamation of the hyperkeratotic layer, giving a false impression of clinical resolution despite persistent dysplastic cells in the basal layer [47, 48]. Conversely, the higher recurrence risk in non-smokers may reflect the inherently more aggressive nature of idiopathic leukoplakia, including a higher proportion of non-homogeneous lesions, upregulated antioxidant defenses (e.g., Nrf2/GSH), and distinct immune microenvironments [11, 49–51]. These factors, together with residual confounding by lesion site and histologic grade [11, 52], warrant cautious interpretation of our findings.

Following all PDT sessions in this study, only low-grade adverse reactions were observed, including mild to moderate pain, hyperaemia, and ulceration. These reactions, which occurred within days post-procedure and were common among patients, were successfully managed with topical symptomatic treatment within one to two weeks. When the above-mentioned conditions occur, symptomatic medication is usually administered. No severe adverse events were recorded. Therefore, ALA-PDT demonstrates a reliable safety profile in the treatment of OLK.

Despite the novel findings of this study, we acknowledge several inherent limitations due to its retrospective, single-arm, target-value design. The absence of concurrent placebo or other treatment controls restricts the strength of our conclusions. Furthermore, although the sample size was calculated based on the Percentage Reduction in Area (%RA) as the primary outcome, it became relatively insufficient for the exploratory objectives of analyzing influencing factors for ITR, TR, and recurrence, which involved 10 to 12 variables. Moreover, the decision to use CO₂ laser pretreatment was based on the clinician’s clinical experience and judgment without a standardized protocol, which constitutes a limitation of this study. Additionally, due to the retrospective nature of this study, we only had access to 1–2 representative slides per case for epithelial thickness measurement, and systematic assessment of intra-lesional regional variation was not feasible. Although this approach follows routine diagnostic practice, it may have introduced some degree of measurement bias. Furthermore, due to the retrospective design, a standardized assessment protocol for defining malignant transformation signs was not pre-specified; however, all evaluations were conducted by the same group of senior clinicians, ensuring consistency. Therefore, the conclusions drawn from these exploratory analyses should be interpreted with caution. We look forward to future large-scale, high-quality randomized controlled trials to further investigate this issue, with the aim of optimizing clinical strategies for PDT in treating OLK and improving outcomes for patients.

## Conclusion

ALA-PDT demonstrates favorable initial and short-term efficacy for OLK, yet recurrence remains a challenge. Lesions with epithelial thickness ≥ 400 μm were associated with numerically higher initial and short-term response rates, although these differences were not statistically significant; however, lesions with epithelial thickness ≥ 400 μm also constituted an independent risk factor for recurrence. Additionally, non-smoking status and lesions in dangerous regions are associated with higher recurrence rates. These findings raise the possibility that integrating epithelial thickness and ITR into clinical practice could aid in risk stratification and personalized long-term management after PDT, although prospective studies are needed to confirm these observations.

## Supplementary Information

Supplementary Material 1.

Supplementary Material 2.

## Data Availability

The data supporting this study’s findings are available from the corresponding author upon reasonable request.

## Abbreviations

OLK: Oral leukoplakia
OPMDs: Oral potentially malignant disorders
PDT: Photodynamic therapy
5-ALA: 5-Aminolevulinic acid
PpIX: Protoporphyrin IX
%RA: Percentage reduction in area
IQR: Interquartile range
ITR: Initial total response
CR: Complete response;
PR: Partial response
NR: No response
TR: Total response
HR: Hazard ratio
MT: Malignant transformation
SD: Standard deviation

## References

[1] Chen Q, Dan H, Pan W, Jiang L, Zhou Y, Luo X, et al. Management of oral leukoplakia: a position paper of the Society of Oral Medicine, Chinese Stomatological Association. Oral Surg Oral Med Oral Pathol Oral Radiol. 2021;132(1):32–43. 34006487 10.1016/j.oooo.2021.03.009

[2] Gates JC, Abouyared M, Shnayder Y, Farwell DG, Day A, Alawi F, et al. Clinical management update of oral leukoplakia: a review from the american head and neck society cancer prevention service. Head Neck. 2025;47(2):733–41. 39584361 10.1002/hed.28013 PMC11717973

[3] Warnakulasuriya S, Ariyawardana A. Malignant transformation of oral leukoplakia: a systematic review of observational studies. J Oral Pathol Med. 2016;45(3):155–66. 26189354 10.1111/jop.12339

[4] Zhang C, Li B, Zeng X, Hu X, Hua H. The global prevalence of oral leukoplakia: a systematic review and meta-analysis from 1996 to 2022. BMC Oral Health. 2023;23(1):645. 37670255 10.1186/s12903-023-03342-y PMC10481497

[5] Warnakulasuriya S, Kujan O, Aguirre-Urizar JM, Bagan JV, González-Moles M, Kerr AR, et al. Oral potentially malignant disorders: A consensus report from an international seminar on nomenclature and classification, convened by the WHO Collaborating Centre for Oral Cancer. Oral Dis. 2021;27(8):1862–80. 33128420 10.1111/odi.13704

[6] Bhattarai BP, Singh AK, Singh RP, Chaulagain R, Søland TM, Hasséus B, et al. Recurrence in oral leukoplakia: a systematic review and meta-analysis. J Dent Res. 2024;103(11):1066–75. 39290142 10.1177/00220345241266519 PMC11504345

[7] Wils LJ, Poell JB, Brink A, Evren I, Brouns ER, de Visscher J, et al. Elucidating the genetic landscape of oral leukoplakia to predict malignant transformation. Clin Cancer Res. 2023;29(3):602–13. 36449687 10.1158/1078-0432.CCR-22-2210

[8] Hopper C. Photodynamic therapy: a clinical reality in the treatment of cancer. Lancet Oncol. 2000;1:212–9. 11905638 10.1016/s1470-2045(00)00166-2

[9] Kataoka H, Nishie H, Tanaka M, Sasaki M, Nomoto A, Osaki T, et al. Potential of photodynamic therapy based on sugar-conjugated photosensitizers. J Clin Med. 2021;10(4):841. 33670714 10.3390/jcm10040841 PMC7922816

[10] Chen Q, Dan H, Tang F, Wang J, Li X, Cheng J, et al. Photodynamic therapy guidelines for the management of oral leucoplakia. Int J Oral Sci. 2019;11(2):14. 30971683 10.1038/s41368-019-0047-0 PMC6458125

[11] Wang Y, Tang H, Wang K, Zhao Y, Xu J, Fan Y. Clinical evaluation of photodynamic therapy for oral leukoplakia: a retrospective study of 50 patients. BMC Oral Health. 2024;24(1):9. 38172857 10.1186/s12903-023-03791-5 PMC10765792

[12] Song Y, Tang F, Liu J, Yang D, Wang J, Luo X, et al. A complete course of photodynamic therapy reduced the risk of malignant transformation of oral leukoplakia. Photodiagnosis Photodyn Ther. 2024;49:104338. 39313101 10.1016/j.pdpdt.2024.104338

[13] Li C, Wang D, Ding J. Potential of photodynamic therapy as a minimally invasive treatment for oral verrucous carcinoma. Photodiagnosis Photodyn Ther. 2024;49:104320. 39208921 10.1016/j.pdpdt.2024.104320

[14] Jing Y, Shu R, Wu T, Liu D, Luo X, Sun J, et al. Clinical efficacy of photodynamic therapy of oral potentially malignant disorder. Photodiagnosis Photodyn Ther. 2024;46:104026. 38403144 10.1016/j.pdpdt.2024.104026

[15] Wang F, Song Y, Xu H, Liu J, Tang F, Yang D, et al. Prediction of the short-term efficacy and recurrence of photodynamic therapy in the treatment of oral leukoplakia based on deep learning. Photodiagnosis Photodyn Ther. 2024;48:104236. 38851310 10.1016/j.pdpdt.2024.104236

[16] van der Waal I, Schepman KP, van der Meij EH, Smeele LE. Oral leukoplakia: a clinicopathological review. Oral Oncol. 1997;33(5):291–301. 9415326 10.1016/s1368-8375(97)00002-x

[17] Chen HM, Yu CH, Tu PC, Yeh CY, Tsai T, Chiang CP. Successful treatment of oral verrucous hyperplasia and oral leukoplakia with topical 5-aminolevulinic acid-mediated photodynamic therapy. Lasers Surg Med. 2005;37(2):114–22. 16037967 10.1002/lsm.20214

[18] Searle T, Al-Niaimi F, Ali FR. Enhancing efficacy of photodynamic therapy with pretreatment. Dermatol Ther. 2020;33(6):e14129. 32761754 10.1111/dth.14129

[19] Bay C, Lerche CM, Ferrick B, Philipsen PA, Togsverd-Bo K, Haedersdal M. Comparison of Physical Pretreatment Regimens to Enhance Protoporphyrin IX Uptake in Photodynamic therapy: a randomized clinical trial. JAMA Dermatol. 2017;153(4):270–8. 28146245 10.1001/jamadermatol.2016.5268

[20] Dong B, He M, Zhang J, Tan Y, Chen X, Wang F, et al. Effect of the combination of diode laser ablation with photodynamic therapy for oral leukoplakia with epithelial dysplasia: a retrospective study. Oral Surg Oral Med Oral Pathol Oral Radiol. 2025;140(2):177–85. 40254477 10.1016/j.oooo.2025.03.003

[21] Luo R, Wang Y, Li R, Ma Y, Chen H, Zhang J, et al. Laser therapy decreases oral leukoplakia recurrence and boosts patient comfort: a network meta-analysis and systematic review. BMC Oral Health. 2024;24(1):469. 38632580 10.1186/s12903-024-04179-9 PMC11025167

[22] Lv J, Xu LX, Li ZX, Lin L, Wu CF, Quan TQ, et al. Longitudinal on-treatment circulating tumor DNA as a biomarker for real-time dynamic risk monitoring in cancer patients: The EP-SEASON study. Cancer Cell. 2024;42(8):1401-1414.e4. 39059389 10.1016/j.ccell.2024.07.001

[23] Raza A, Khan AQ, Inchakalody VP, Mestiri S, Yoosuf Z, Bedhiafi T, et al. Dynamic liquid biopsy components as predictive and prognostic biomarkers in colorectal cancer. J Exp Clin Cancer Res. 2022;41(1):99. 35292091 10.1186/s13046-022-02318-0 PMC8922757

[24] Gyselinck I, Huts H, De Vos M, Janssens W. Time T-to understand treatment response. Lancet Respir Med. 2025;13(6):482–4. 40319904 10.1016/S2213-2600(25)00118-3

[25] Nautiyal M, Kumar Vadivel J, Ramalingam K. Prevalence of keratosis in the oral cavity: a clinical retrospective study. Cureus. 2024;16(1):e52199. 38347988 10.7759/cureus.52199 PMC10859883

[26] Harris P, Bissonnette C, Tabet P, Wittmer R. Common white lesions of the oral cavity: review of clinical presentations and management. Can Fam Physician. 2025;71(1):19–25. 39843200 10.46747/cfp.710119 PMC11753272

[27] Warnakulasuriya S, Johnson NW, van der Waal I. Nomenclature and classification of potentially malignant disorders of the oral mucosa. J Oral Pathol Med. 2007;36(10):575–80. 17944749 10.1111/j.1600-0714.2007.00582.x

[28] Tenore G, Mohsen A, Fantozzi PJ, Golrang A, Podda GM, Rocchetti F, et al. Risk assessment for malignant transformation in patients with oral proliferative leukoplakia: a 10-year retrospective cohort study. Cancers (Basel). 2025;18(1):2. 41514515 10.3390/cancers18010002 PMC12785022

[29] Tovaru S, Costache M, Perlea P, Caramida M, Totan C, Warnakulasuriya S, et al. Oral leukoplakia: a clinicopathological study and malignant transformation. Oral Dis. 2023;29(4):1454–63. 34982498 10.1111/odi.14123

[30] Di Stasio D, Romano A, Russo D, Fiori F, Laino L, Caponio VCA, et al. Photodynamic therapy using topical toluidine blue for the treatment of oral leukoplakia: a prospective case series. Photodiagnosis Photodyn Ther. 2020;31:101888. 32593778 10.1016/j.pdpdt.2020.101888

[31] Piattelli A, Fioroni M, Santinelli A, Rubini C. Bcl-2 expression and apoptotic bodies in 13-cis-retinoic acid (isotretinoin)-topically treated oral leukoplakia: a pilot study. Oral Oncol. 1999;35(3):314–20. 10621853 10.1016/s1368-8375(98)00095-5

[32] Hong WK, Endicott J, Itri LM, Doos W, Batsakis JG, Bell R, et al. 13-cis-retinoic acid in the treatment of oral leukoplakia. N Engl J Med. 1986;315(24):1501–5. 3537787 10.1056/NEJM198612113152401

[33] Lippman SM, Batsakis JG, Toth BB, Weber RS, Lee JJ, Martin JW, et al. Comparison of low-dose isotretinoin with beta carotene to prevent oral carcinogenesis. N Engl J Med. 1993;328(1):15–20. 8416267 10.1056/NEJM199301073280103

[34] Gu J, Liao J, Zhang T, Zhang Y, Shepherd S, Macluskey M, et al. Quantitative characterization of undulation patterns and regional features of the oral basement membrane zone using optical coherence tomography. J Biophotonics. 2025;18(10):e202500252. 40650378 10.1002/jbio.202500252

[35] Stasio DD, Lauritano D, Iquebal H, Romano A, Gentile E, Lucchese A. Measurement of oral epithelial thickness by optical coherence tomography. Diagnostics (Basel). 2019;9(3):90. 31390841 10.3390/diagnostics9030090 PMC6787684

[36] Li Y, Wang B, Zheng S, He Y. Photodynamic therapy in the treatment of oral leukoplakia: A systematic review. Photodiagnosis Photodyn Ther. 2019;25:17–22. 30391342 10.1016/j.pdpdt.2018.10.023

[37] Pietruska M, Sobaniec S, Bernaczyk P, Cholewa M, Pietruski JK, Dolińska E, et al. Clinical evaluation of photodynamic therapy efficacy in the treatment of oral leukoplakia. Photodiagnosis Photodyn Ther. 2014;11(1):34–40. 24211597 10.1016/j.pdpdt.2013.10.003

[38] Kawczyk-Krupka A, Waśkowska J, Raczkowska-Siostrzonek A, Kościarz-Grzesiok A, Kwiatek S, Straszak D, et al. Comparison of cryotherapy and photodynamic therapy in treatment of oral leukoplakia. Photodiagnosis Photodyn Ther. 2012;9(2):148–55. 22594985 10.1016/j.pdpdt.2011.12.007

[39] Yuan Y, Jiang P, Xiao C, Lei J, Cheng B, Hu H, et al. Oxygen-boosted dual-section microneedle patch for enhanced drug penetration and improved photodynamic and anti-inflammatory therapy in psoriasis. Acta Pharm Sin B. 2026;16(1):458–69. 41584337 10.1016/j.apsb.2025.09.037 PMC12827881

[40] Jung E, Kwon S, Song N, Kim N, Jo H, Yang M, et al. Tumor-targeted redox-regulating and antiangiogenic phototherapeutics nanoassemblies for self-boosting phototherapy. Biomaterials. 2023;298:122127. 37086554 10.1016/j.biomaterials.2023.122127

[41] Li N, Dong F, Sun L, Qian Y, Zhang L, Wang G, et al. Carrier-free delivery of nucleic acid and photosensitizer nanoparticles for enhanced photodynamic and gene antitumour therapy. Fundam Res. 2024;5(4):1698–709. 40777773 10.1016/j.fmre.2024.03.014 PMC12327862

[42] Xu T, Zhong L, Liu Q, Wang F, Kuang W, Liang J, et al. NRF2 modulates WNT signaling pathway to enhance photodynamic therapy resistance in oral leukoplakia. EMBO Mol Med. 2025;17(7):1794–824. 40494896 10.1038/s44321-025-00256-w PMC12254380

[43] Guan JY, Luo YH, Lin YY, Wu ZY, Ye JY, Xie SM, et al. Malignant transformation rate of oral leukoplakia in the past 20 years: A systematic review and meta-analysis. J Oral Pathol Med. 2023;52(8):691–700. 37224426 10.1111/jop.13440

[44] Chen XN, Wang ZR, Jin TH, Zhou ZT, Li CX, Shi LJ. Risk factors associated with oral leukoplakia: a cross-sectional study of 430 patients. Zhonghua Kou Qiang Yi Xue Za Zhi. 2025;60(7):731–8. 40619137 10.3760/cma.j.cn112144-20241215-00483

[45] van der Waal I. Potentially malignant disorders of the oral and oropharyngeal mucosa; terminology, classification and present concepts of management. Oral Oncol. 2009;45(4–5):317–23. 18674954 10.1016/j.oraloncology.2008.05.016

[46] Liu W, Bao ZX, Shi LJ, Tang GY, Zhou ZT. Malignant transformation of oral epithelial dysplasia: clinicopathological risk factors and outcome analysis in a retrospective cohort of 138 cases. Histopathology. 2011;59(4):733–40. 21916948 10.1111/j.1365-2559.2011.03938.x

[47] Wang F, Shi Y, Dong Y, Liu T, Dan H, Wang J, et al. Photodynamic therapy combined with laser drilling successfully prevents the recurrence of refractory oral proliferative verrucous leukoplakia. Photodiagnosis Photodyn Ther. 2021;36:102564. 34610431 10.1016/j.pdpdt.2021.102564

[48] Sang Z, Zhu T, Qu X, Zhang Z, Wang W, Hao Y. A hyaluronic acid-based dissolving microneedle patch loaded with 5-aminolevulinic acid for improved oral leukoplakia treatment. Colloids Surf B Biointerfaces. 2025;245:114216. 39260274 10.1016/j.colsurfb.2024.114216

[49] Wan W, Gao X, Song S, Peng J, Jiang C, Fang C, et al. 5-aminolevulinic acid photodynamic therapy for extensive oral leukoplakia with concomitant oral submucous fibrosis: A case report. Photodiagnosis Photodyn Ther. 2023;41:103203. 36400168 10.1016/j.pdpdt.2022.103203

[50] Cai X, Zhang J, Peng Y, Yao Z, Huang J, Tang Q, et al. The preliminary exploration of immune microenvironment in oral leukoplakia concomitant with oral submucosal fibrosis: A comparative immunohistochemical study. J Oral Pathol Med. 2023;52(7):666–72. 37084341 10.1111/jop.13434

[51] Yang D, Yang D, Song Y, Liu J, Wang Y, Feng X, et al. ferroptosis induction enhances photodynamic therapy efficacy for OLK. J Dent Res. 2024;103(12):1227–37. 39394822 10.1177/00220345241280257 PMC11653335

[52] Sulewska M, Wisniewski P, Stepniewska M, Poloczek Z, Chodzinski D, Melion P, et al. Efficacy of 5-ALA photodynamic therapy in dysplastic oral leukoplakia: systematic review and meta-analysis. Pharmaceutics. 2026;18(2):254. 41754995 10.3390/pharmaceutics18020254 PMC12944706

