# Supplementary material for: Epithelial thickness as a new predictor of recurrence in oral leukoplakia after photodynamic therapy: a retrospective cohort study
Source: BMC Oral Health. 2026 Jun 11;26:1480. doi: 10.1186/s12903-026-08832-3 (PMC13483677; doi:10.1186/s12903-026-08832-3)
Supplement: Supplementary file 1 [file 12903_2026_8832_MOESM1_ESM.docx]

**Table S1. Histopathological Characteristics of the Study Cohort**

| **Histopathological Feature** | **n (%)** | ***p*-value (Univariate analysis)** | | |
| --- | --- | --- | --- | --- |
|  |  | **ITR** | **TR** | **Recurrence** |
| **Hyperkeratosis** |  | **0.494** | **0.065** | **0.055** |
| Yes | 40 (66.7%) |  |  |  |
| No | 20 (33.3%) |  |  |  |
| **Acanthosis** |  | **0.259** | **0.434** | **0.670** |
| Yes | 13 (21.7%) |  |  |  |
| No | 47 (78.3%) |  |  |  |
| **Epithelial Dysplasia** |  | **0.362** | **0.824** | **0.742** |
| No | 2 (3.3%) |  |  |  |
| Mild | 15 (25.0%) |  |  |  |
| Moderate | 31 (51.7%) |  |  |  |
| Severe | 12 (20.0%) |  |  |  |
| **Inflammatory Changes** |  | **0.299** | **0.373** | **0.416** |
| Yes | 27 (45%) |  |  |  |
| No | 33 (55%) |  |  |  |
| **Epithelial thickness (μm) ^a^** |  | **0.256** | **0.328** | **0.030 *** |
| ≥ 400μm | 31 (58.5%) |  |  |  |
| < 400μm | 22 (41.5%) |  |  |  |

^a^ the sample size for the epithelial thickness model is n=53 after listwise deletion of 7 cases with missing values.

^⁎^ a significant statistical difference was observed, *p* < 0.05.
