# Supplementary material for: Epithelial thickness as a new predictor of recurrence in oral leukoplakia after photodynamic therapy: a retrospective cohort study
Source: BMC Oral Health. 2026 Jun 11;26:1480. doi: 10.1186/s12903-026-08832-3 (PMC13483677; doi:10.1186/s12903-026-08832-3)
Supplement: Supplementary file 2 [file 12903_2026_8832_MOESM2_ESM.docx]

**Table S2. ITR, short-term efficacy, and recurrence outcomes by grade of epithelial dysplasia (N=60) ^a^**

|  | **ITR** | **short-term efficacy** | | | **Recurrence** |
| --- | --- | --- | --- | --- | --- |
|  |  | **CR** | **PR** | **NR** |  |
| **Epithelial dysplasia** | |  |  |  |  |
| No | **2/2**  (100%) | **0/2**  (0%) | **2/2**  (100%) | **0/2**  (0%) | **1/2**  (50%) **^b^** |
| Mild | **9/15**  (60%) | **3/15**  (20%) | **9/15**  (60%) | **3/15**  (20%) | **5/12**  (41.7%) **^b^** |
| Moderate | **25/31**  (80.6%) | **10/31**  (32.3%) | **19/31**  (61.3%) | **2/31**  (6.4%) | **14/29**  (48.3%) |
| Severe | **11/12**  (91.7%) | **5/12**  (41.7%) | **6/12**  (50%) | **1/12**  (8.3%) | **6/11**  (54.5%) **^b^** |
| **Total, n (%)** | **47/60**  (78.3%) | **18/60**  (30%) | **36/60**  (60%) | **6/60**  (10%) | **26/54**  (48.1%) **^b^** |

^a^ Data shown are for the full cohort (N=60), in contrast to Tables 2/3/4 which are based on the subset with complete epithelial thickness data (7 missing cases).

**^b^** Because recurrence is not applicable to the 6 NR patients, the denominator for calculating the recurrence rate in each group was adjusted by subtracting the corresponding number of NR patients.
